# Automatic analysis of the continuous edges of stone tools reveals fundamental handaxe variability

**DOI:** 10.1038/s41598-024-57450-y

**Published:** 2024-03-28

**Authors:** Antoine Muller, Gonen Sharon, Leore Grosman

**Affiliations:** 1 Computational Archaeology Laboratory, Institute of Archaeology, Hebrew University of Jerusalem, Jerusalem, Israel; 2 Multidisciplinary Studies, Tel-Hai College, East Campus, Upper Galilee, Israel

**Keywords:** Handaxe, Biface, Lower Palaeolithic, Acheulean, 3D lithic analysis, Edge angle, Archaeology, Archaeology

## Abstract

The edges of stone tools have significant technological and functional implications. The nature of these edges–their sharpness, whether they are concave or convex, and their asymmetry–reflect how they were made and how they could be used. Similarly, blunt portions of a tool’s perimeter hint at how they could have been grasped or hafted and in which directions force could be applied. However, due to the difficulty in accurately measuring the complex 3D geometry of tool edges with traditional methods, their attributes are often overlooked. When they are analyzed, they have traditionally been assessed with visual qualitative categories or unreliable physical measurements. We introduce new computational 3D methods for automatically and repeatably measuring key attributes of stone tool edges. These methods allow us to automatically identify the 3D perimeter of tools, segment this perimeter according to changes in edge angles, and measure these discrete edge segments with a range of metrics. We test this new computational toolkit on a large sample of 3D models of handaxes from the later Acheulean of the southern Levant. Despite these handaxes being otherwise technologically and morphologically similar, we find marked differences in the amount of knapped outline, edge angle, and the concavity of their edges. We find many handaxes possess blunt portions of perimeter, suitable for grasping, and some handaxes even possess more than one discrete sharp edge. Among our sample, sites with longer occupations and more diverse toolkits possessed handaxes with more diverse edges. Above all, this paper offers new methods for computing the complex 3D geometry of stone tool edges that could be applied to any number of artifact types. These methods are fully automated, allowing the analysis and visualization of entire assemblages.

## Introduction

The geometry of tool edges plays a significant role in tool-use for both lithic^1–8^ and non-lithic tools^9^. The length, angle, and shape of edges affect functionality by influencing how tools are grasped, as well as the amount and direction of force applied during use. For handaxes, Key and colleagues found that while their size and shape exerted minimal influence on functional efficiency^10^, edge angle played a much more important role^11^. Moreover, the portions of edge that were knapped into sharp edges versus those that remained blunt likely reflect the technological choices of past knappers.

Although the geometry of tool edges is fundamental to understanding functional and technological variability, handaxe edges remain sorely understudied. This is likely owing to the difficulty of reliably measuring key edge properties such as length, angle, concavity, and asymmetry. These features are typically estimated with inaccurate manual measurements or by qualitative categories. Some have begun using 3D models of artifacts to help with the measurement of these attributes^12–15^, but these measurements are taken manually from the 3D models at user-defined points. As of now, we even lack a reliable means of identifying handaxe edges on 3D scans at all. For handaxes that are sharp around their entire perimeter, this may seem trivial. But for those with blunt portions, their edge can be ambiguous. If blunt portions of handaxe perimeter are not considered an ‘edge’, then what angle is considered ‘blunt’? How sharp is sharp enough to constitute an edge? Is a mean edge angle for each tool sufficient? Or should distinct portions of edges with noticeably different edge angles be analyzed separately? 3D analyzes of handaxe edge geometry can offer objective and repeatable ways of answering these questions.

### Case study: later Acheulean of the southern Levant

To identify previously overlooked variability in handaxe edge geometry and to begin answering these questions, we require a well-controlled sample of similarly made handaxes. Those from the later Acheulean of the southern Levant provide a large sample of technologically and morphologically similar handaxes, which are, with rare exception, made on local flint^16–18^. Herzlinger et al.^19^ found that handaxes from later Acheulean sites were morphologically similar to each other when compared to Early and Middle Acheulean sites. Importantly also, many have noted that handaxes in the later Acheulean southern Levant frequently possess portions that are un-knapped and/or blunt^20–25^. This period and region is thus ideal for testing our new methods aimed at identifying subtle variability in handaxe edges. We analyze 3D models of handaxes from Ma’ayan Barukh, Jaljulia, Holon, Revadim, and Nahal Zihor, sites which offer large sample sizes of morphologically similar handaxes, all made on flint, and all from the later Acheulean of the southern Levant. We hypothesize that yet unmeasured variability in edge geometry will identify key inter-site variability among these five sites. If any previously overlooked variability related to edge geometry can be found, we aim to explore the possible explanations for this inter-site variability.

### Outline detection

**Figure 1:**
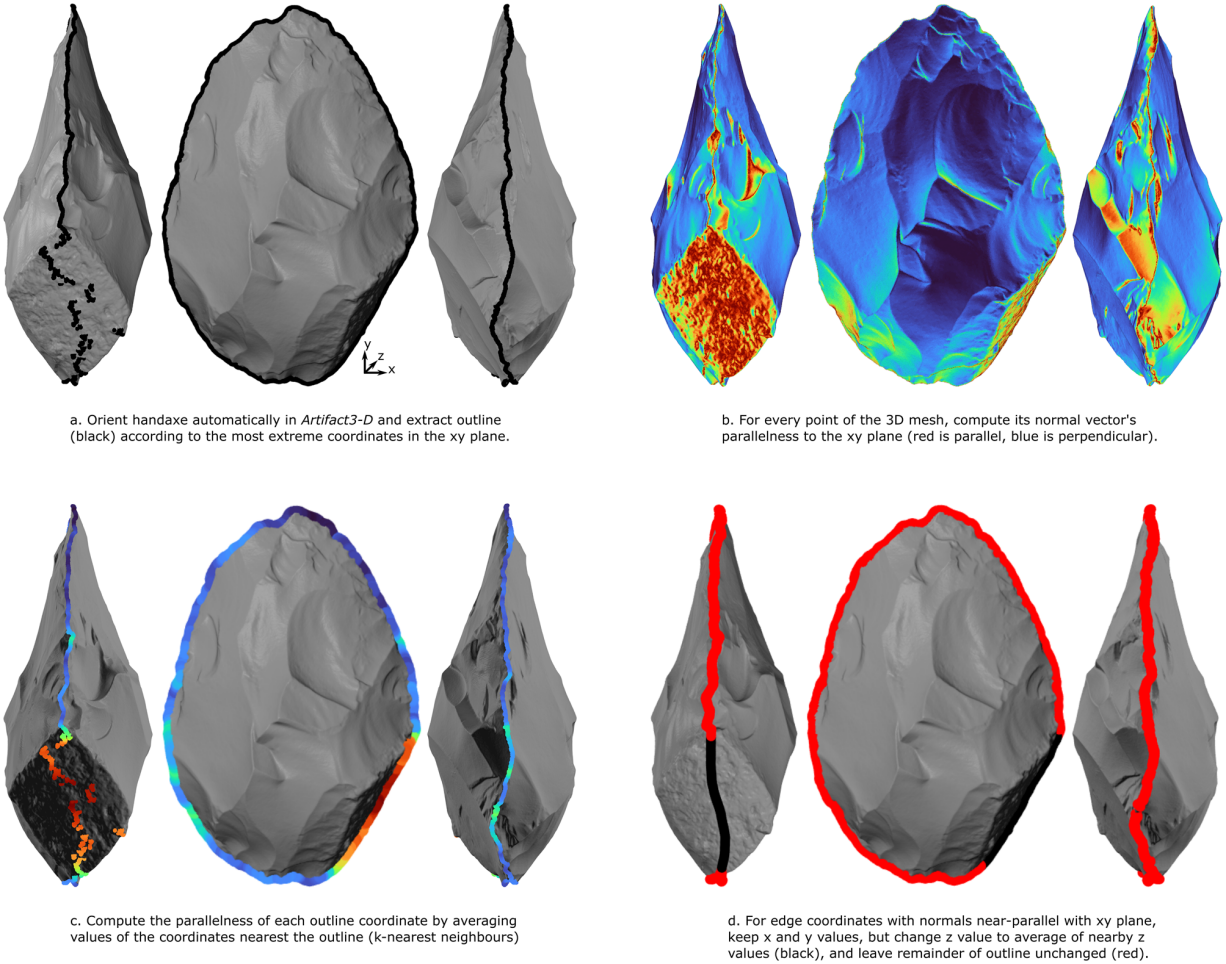
Automatic edge detection method. (**a**) The 3D outline produced by projecting the scan onto the x–y plane, showing how the sharp edges of handaxes are easily identified, but blunt portions of handaxe perimeter require further processing. (**b**) The 3D mesh of the example handaxe, colored according to how parallel each normal vector is to the x–y plane, showing how blunt portions of perimeter are identified. (**c**) The same values as in b but averaged for the coordinates in a k-nearest neighborhood to the perimeter. (**d**) The fixed coordinates of the outline shown in black, made by averaging the z coordinates of this blunt region.

Before variability in handaxe edge geometry can be quantified, a reliable and fair way of separating a handaxe into its two faces is needed. The geometric properties of handaxe 3D models can be used to automatically identify their 3D perimeter. See Fig. 1 for a visual summary of the outline detection method and the Methods section for a more detailed description. With this 3D outline, we can then measure the edge angle at every coordinate of this outline, segment the outline according to these angle values, and then measure the length, transverse asymmetry, and surface concavity of these segments. We briefly summarize each of these steps here and explore previous attempts at quantifying tool edges.

### Edge angle measurement

Due to the complex geometry of handaxe edges, comprised of many intersecting flake scars, edge attributes are difficult to determine and thus typically involve inaccurate manual measurements or qualitative visual assessments. For instance, lithic edge angles are notoriously difficult to measure^26^. Recent methods for quantifying edge angles use 3D scans to measure 2D cross sections^12,15,27–32^, or normal vectors surrounding a user-defined coordinate^33,34^. Even methods that measure angles at every coordinate of the edge^8,35–37^ do so at a user-defined distance from each of these coordinates. Taking edge angle measurements with any user-chosen coordinate oversimplifies the complex geometry of tool edges, as edge angle values change continuously between any two coordinates along the edge of the handaxe, as well as between a coordinate on the edge and one on the handaxe’s surface.

**Figure 2:**
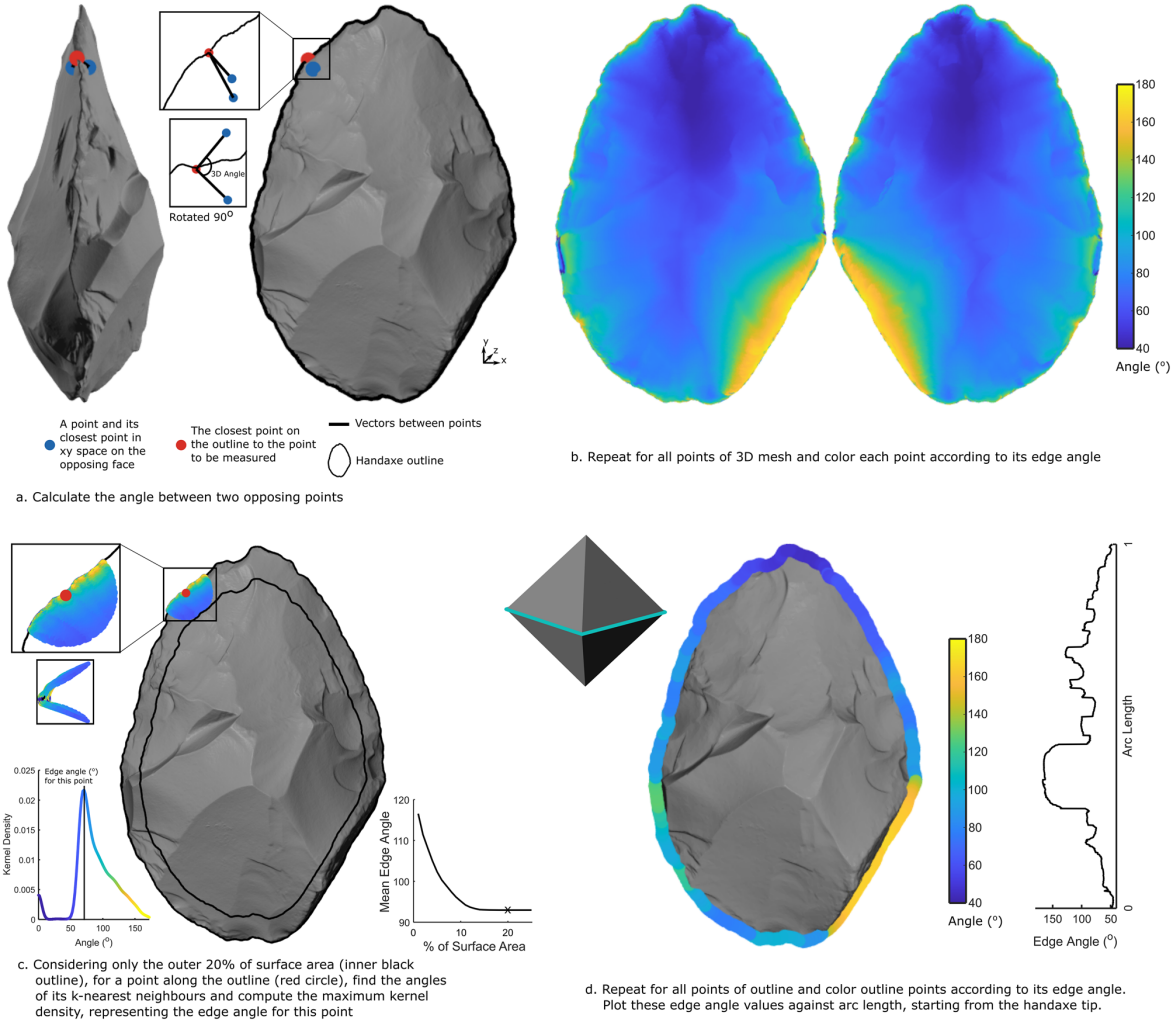
Continuous Edge Angle Measurement (CEAM) method. (**a**) An example of the three coordinates used to measure the edge angle value of an individual coordinate of the 3D mesh. (**b**) The result of repeating this process for every coordinate of the mesh. (**c**) The method for calculating the edge angle for every coordinate of the handaxe’s outline, while excluding outlier angle values using the maximum kernel density value. (**d**) The result of repeating this process for every coordinate of the outline, generating continuous edge angle values around the entire perimeter of the handaxe. Inset at the top left of d is the result of running the CEAM analysis on a regular octahedron, showing that the edge angle value of 109.47° matches precisely its known dihedral angle.

Valletta et al.^7^ introduced a reliable 3D edge angle measurement that is available as a function in the *Artifact3-D* software^38^, addressing these issues of user-defined position and fixed depth of measurements. This method was developed with blades and backed blades in mind and is best suited to discrete portions of edges divided by a small number of ridges delineated by user-selected points along the edge. Handaxes however, are comprised of one long intersection between two faces, with each flake scar on this intersection contributing both convexities and concavities, which in turn alters the edge angle around the handaxe perimeter. The complex geometry of handaxe edges necessitates a new continuous method of angle measurement that relies on an automatically identified outline without user-input. Thus, we introduce the Continuous Edge Angle Measurement (CEAM – pronounced ‘seam’) which automatically computes the edge angle at every coordinate of the tool’s edge (Fig. 2).

### Edge segmentation

Kleindienst’s^39^ formative definition of handaxes specified that handaxes are “characterized by a cutting edge around the entire circumference of the tool, or more *rarely* around the entire circumference with the exception of the butt” (emphasis added). After much subsequent research on handaxes, it appears now that handaxes with blunt bases may not be such a rare occurrence^40^. These blunt portions of handaxe perimeter are routinely conceptualized as areas suited for grasping^11,15,32,41–44^, while the sharp portions of handaxes are generally conceived as the functional area^5,45^ and have been associated with tasks like butchery and woodwork^3,46–49^. Thus, we define handaxe edges as the ‘sharp’ portions of their perimeter, distinct from ‘blunt’ portions. One of the first tasks of this analysis is to find an objective way of identifying which edge angles can be considered sharp enough to constitute an ‘edge’ among our sample.

Tools possessing portions of sharp edge with noticeably different edge angles have previously led to the identification of bifaces with more than one use-edge^15,43,50–53^. A tool with two or more portions of perimeter with drastic discontinuities in edge angle values along its perimeter precludes it from an optimal continuous use-motion. These different edges could even have served different functions. Thus, we aim to not only distinguish between sharp (edge) and blunt (non-edge) portions of handaxe perimeter, but to also segment portions of the sharp edge into discrete segments.

**Figure 3:**
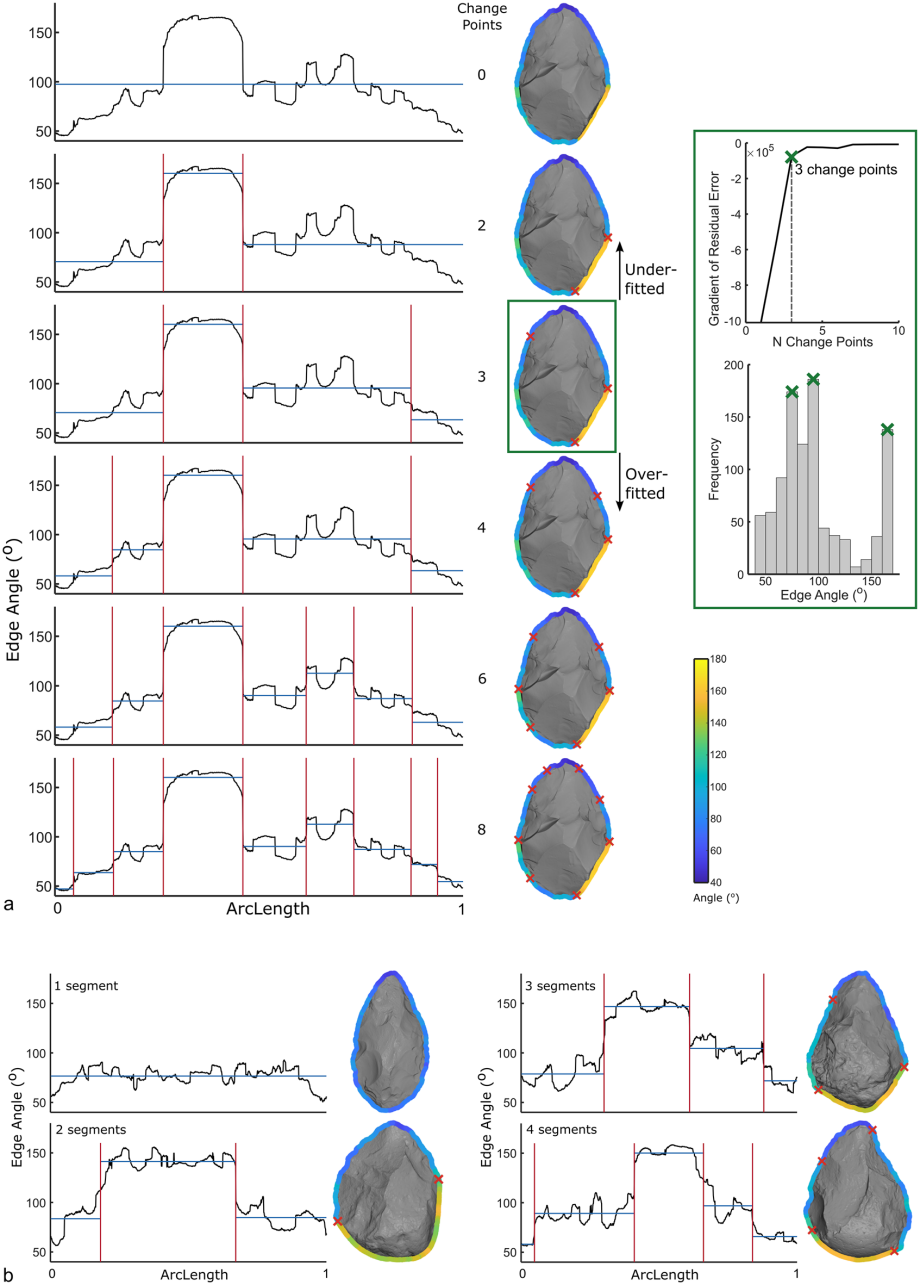
Automatic edge segmentation method using change point detection. (**a**) One example handaxe with different numbers of change points applied. Plots of arc length versus edge angle are shown on the left. Vertical red lines represent change points and horizontal blue lines represent the local mean of each segment. The method of automatically choosing how many change points to apply is shown on the right. For this example, the elbow of the residual error plot indicates that three change points should be applied. (**b**) Other example handaxes with different numbers of segments.

Previous attempts at segmenting handaxes into discrete portions have divided them into arbitrary sectors^54–61^. Others divide handaxe edges according to the location of cortex^62^, the nature of retouch^22^, or the sharper portions of perimeter^51,63,64^. However, these determinations are typically made subjectively. The techno-functional method involves a much more detailed analysis of these edges and segments handaxes^12,15,42–44,65,66^ and other tools^13,14,50,67–73^ into active versus prehensile units, but these analyzes remain subjective also. Here, we aim to offer objective means of segmenting the edges of handaxes according to significant changes in their edge angle values (Fig. 3).

### Edge measurement

Once the outlines of handaxes can be automatically segmented, we seek to measure these segments. Aside from edge angle, we also need new ways of reliably measuring a range of edge variables. For instance, edge length is fundamental to understanding how much usable edge each artifact possesses. The edge length of lithic artifacts has previously been estimated via manual linear measurements^74,75^ or 2D measurements in image processing software^76–79^. We seek a new method to quantify this length automatically and objectively in 3D and to compute the proportion of sharp versus non-sharp edge. Other attributes, like edge asymmetry and concavity, are fundamental to techno-functional analyzes and are usually assessed qualitatively as either symmetrical or asymmetrical^13,22,43,69,80^, and as any combination of convex, planar, or concave^24,42,65,68,69,72,73^. We aim to quantify edge asymmetry and concavity as continuous variables using 3D scans.

It is clear that traditional methods are insufficient to quantify the complexity of handaxe edges. What is needed then, are computational 3D methods of measuring the geometry of handaxe edges around their entire perimeter. Here, we automate and make available a new toolkit of 3D computational metrics for quantifying handaxe edge geometry. These methods first involve loading a 3D model into the *Artifact3-D* program^38^ and choosing an orientation, either an automatic position based on its geometric properties or a user-chosen position. All subsequent steps are conducted entirely automatically using custom MATLAB code developed for this present study^81^. These methods include outline detection, continuous edge angle measurement, edge segmentation according to these angle values, and various edge segment measurements. We test these new methods using a case study of the southern Levantine later Acheulean, with 686 3D scans of handaxes from five sites.

## Results

**Figure 4:**
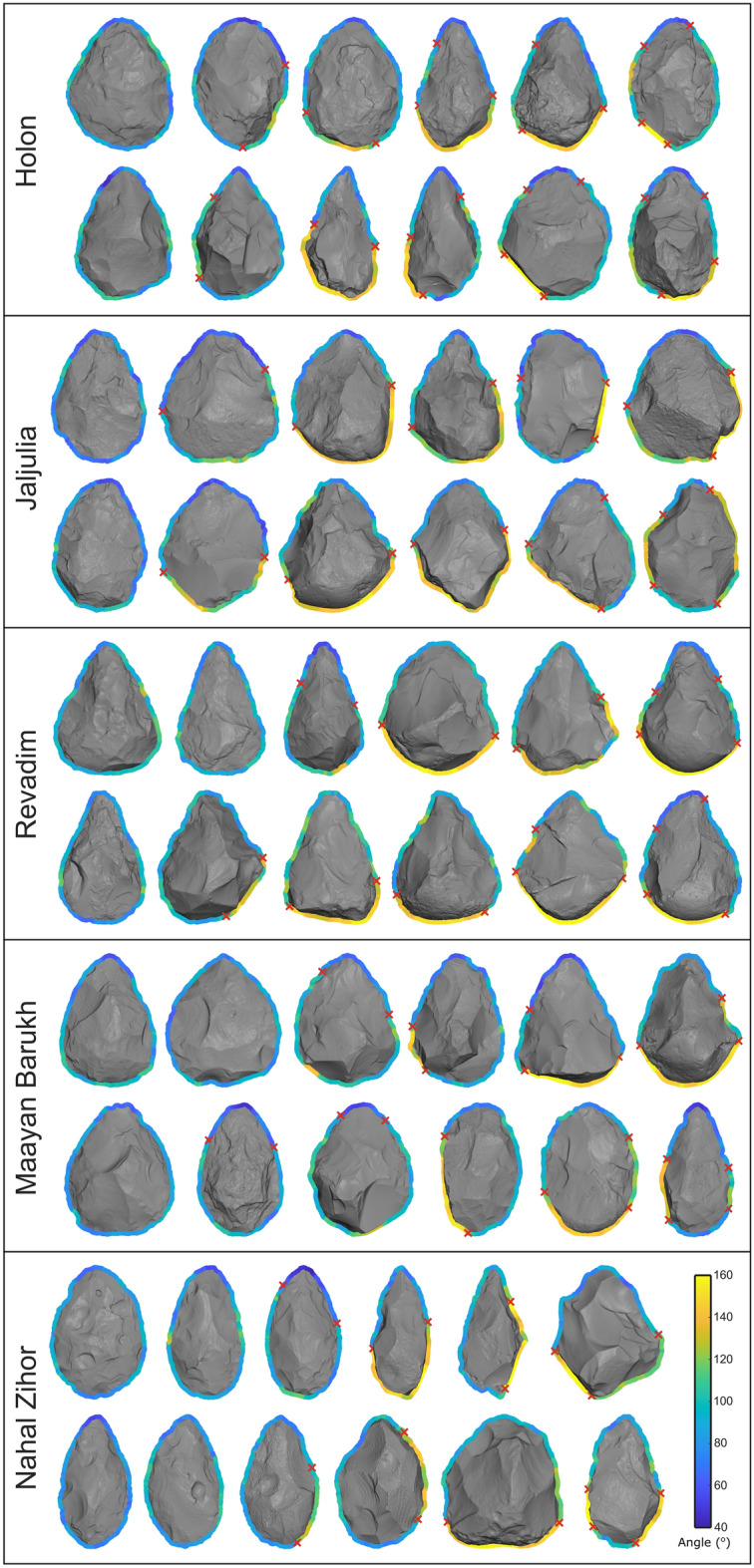
Examples of handaxes from each of the five sites, with the results of the CEAM and edge segmentation analyzes displayed around their outline. Edge angles are displayed with color on the outline. Red crosses represent the border between discrete outline segments as identified by the change points analysis. Handaxes are not to scale and were chosen to visually represent the variability within each site.

The CEAM analysis automatically measures the edge angle at every coordinate of the artifact’s outline and segments this outline where necessary according to these angle values. As examples, Fig. 4 shows 12 handaxes from each site, not to scale, with the output generated by the CEAM analysis, visualizing the diversity of edges within and between each site. Red crosses denote change points, distinguishing discrete edge segments from each other. Even from this visual examination alone, there is much variability in the locations and angles of edge segments. Some handaxes possess sharp edges around their entire perimeter, while others possess significant portions of blunt perimeter. As expected, sharper (blue) edge segments are commonly located near the tip, and blunter (yellow) segments are typically located near the base.

**Figure 5:**
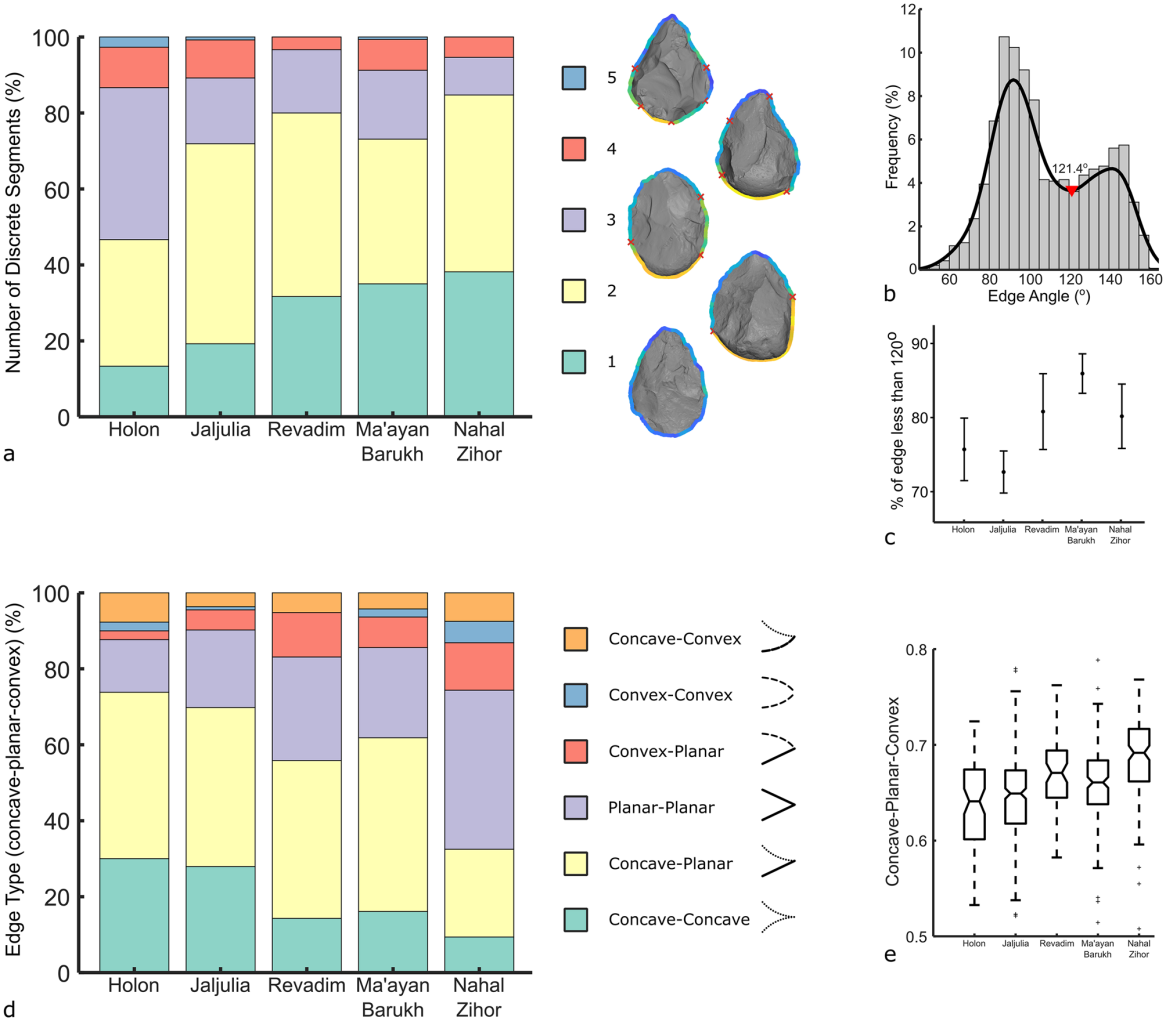
Results of the edge analyzes. (**a**) Stacked bar chart of the counts of discrete edge segments expressed as a percent of all handaxes per site. Examples of handaxes with different numbers of discrete edges are shown on the right. (**b**) Histogram of the edge angle values for all 1445 discrete edge segments. (**c**) Mean and whisker plot of the percent of sharp perimeter (i.e. less than 120°) with standard error bars with a 95% confidence interval. (**d**) Stacked bar chart of the counts of edge type, according to any combination of concave, planar, and convex, expressed as a percent of all handaxes per site. e: Boxplot of concavity values per site, with higher values representing more convexity.

From the 686 handaxes examined in this sample, edge segmentation analysis identified a total of 1445 discrete segments, or an average of 2.1 segments per handaxe. Figure 5a shows the proportion of segments organized by site, showing the intra- and inter-site variability. This and all subsequent charts are ordered left-to-right by the proportion of handaxes possessing only one segment (Holon, Jaljulia, Revadim, Ma’ayan Barukh, Nahal Zihor). A chi-squared test reveals significant differences between the proportions of segment numbers among the five sites (X^2^ = 62.96, d.f. = 16, *p* < 0.001). For example, handaxes from Nahal Zihor possess only one segment at a rate of almost triple that of Holon. At Holon, there are more handaxes with three or more segments than there are with only one or two segments. Both Holon and Jaljulia possess a higher proportion of handaxes with four and five segments than the remainder of the sites.

Overall, there is much inter-site variability in the number of outline segments per handaxe. Thus far however, all discrete segments have been treated equally. But it is clear from the continuous edge angle values that some segments possess much higher values than others (bright yellow). Previous experiments by Key and colleagues found that portions of handaxe perimeter were likely intentionally left blunt for grasping^11^, that the type of grip was influenced by the edge angle of the base^41^, and that sharper tips opposing these grasping areas allowed more force to be imparted^82^. Techno-functional analyses also routinely make a distinction between active and prehensile areas of handaxes, based partly on their edge angles^15,42–44^. Thus, a distinction needs to be made between portions of the outline that are sufficiently sharp for routine use, and those that are blunter, likely lending themselves better to grasping. However, there is no way to distinguish ‘sharp’ from ‘not-sharp’ a priori. Instead of a deductive method, we propose an inductive means of distinguishing these edges based on the frequency of angle values within our sample.

To decide where to place the cut-off between edge and non-edge, we plot a histogram in Fig. 5b of edge angle values for all 1445 segments examined here. This distribution possesses two peaks, divided by a trough at 121.4°. These two peaks represent a cluster of common sharp edge angles and a cluster of common blunt edge angles. We use this trough in the bimodal distribution as an objective a posteriori threshold for separating ‘sharp’ from ‘blunt’ portions of perimeter. For simplicity, we round this threshold down to 120°. Owing to their bifacial nature, the edges of handaxes tend to possess higher edge angles than unifacial items like flakes or flake tools. Thus, this relatively high angle threshold appears reasonable, as a range of tasks could foreseeably be achieved with angles in this higher range. At other sites or regions this bimodal distribution may reveal a different threshold between edge and non-edge. For the remainder of this analysis, any outline segments greater than 120° are treated as blunt portions of handaxe perimeter, likely better suited for grasping, rather than as sharp edge segments. Of the 1445 total outline segments, 961 possess an average angle below 120° and are treated as edges.

Having distinguished sharp from blunt outline segments, we can compare the mean edge angle values of each discrete edge segment per site. A Kruskal–Wallis test (H = 24.05, d.f. = 960 *p* < 0.001) with post hoc, Bonferroni corrected, Mann–Whitney comparisons shows that the Holon (U = 3539, d.f. = 206, p < 0.01) and Ma’ayan Barukh (U = 6527, d.f. = 312, *p* < 0.01) handaxes possess sharper handaxes than those from Revadim, and that the Ma’ayan Barukh handaxes were also sharper than those from Jaljulia (U = 35,809, d.f. = 593, *p* < 0.05).

The 961 edges mostly conform to areas that have been knapped or ‘worked’. Thus, we can compute the proportion of handaxe outlines that have been worked into a sharp edge, shown in Fig. 5c as the percent of edge length less than 120° in a mean and whisker plot with standard error bars with a 95% confidence interval. Overall, the handaxes from Revadim, Nahal Zihor, and especially Ma’ayan Barukh, stand apart from those from Holon and Jaljulia as being more completely reduced around their perimeter. Specifically, the handaxes of Nahal Zihor possess a significantly higher percentage of sharp (< 120°) edge length than those from Jaljulia (U = 13,359, d.f. = 390, *p* < 0.01), while Ma’ayan Barukh handaxes possess significantly more than those from both Jaljulia (U = 13,810, d.f. = 419, *p* < 0.01) and Holon (U = 4156, d.f. = 234, *p* < 0.01).

Thus far, we have considered only the edge angle of these handaxe edges, but a range of other variables can be computed, like the concavity of the edge surfaces. Considering only the 961 sharp edge segments, Fig. 5d shows the significant differences (X^2^ = 104.34, d.f. = 20, *p* < 0.001) in the proportion of edges that are any combination of concave, planar, and convex. Again, differences among the sites occur from left-to-right on the chart. For example, the proportion of concave-concave edges at Revadim, Ma’ayan Barukh, and especially Nahal Zihor, are less than half compared with Jaljulia and Holon. Meanwhile, Nahal Zihor also has considerably more planar-planar edges than any other site. Figure 5e shows similar results when taking mean concavity values for each handaxe as a continuous variable. After a Kruskal–Wallis test (H = 89.23, d.f. = 685, *p* < 0.001), Bonferroni corrected Mann–Whitney tests show that Nahal Zihor handaxes possess edge surfaces with significantly more convexity than all other sites (*p* < 0.05). Holon and Jaljulia are statistically similar for this metric, as are Revadim and Ma’ayan Barukh. But, Revadim and Ma’ayan Baruch are significantly more convex than Holon and Jaljulia (*p* < 0.05).

**Figure 6:**
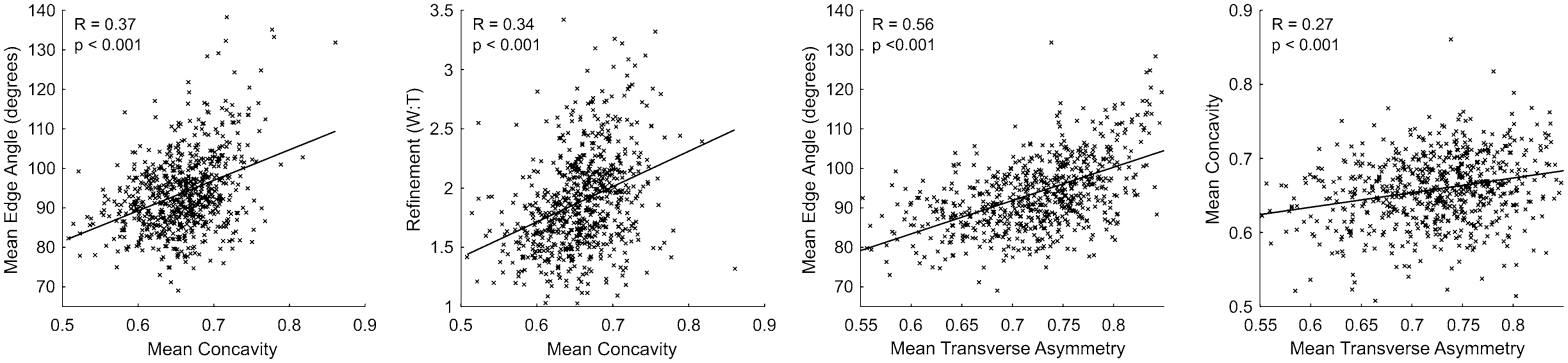
Scatter plots showing the relationship between selected variables.

Lastly, we explore how some of these metrics covary to propose how the variability in concavity/convexity and transverse asymmetry may be explained (Fig. 6). For instance, the convexity of handaxe edge surfaces is positively correlated with edge angle. This is probably explained by the tendency for larger handaxe thinning flakes to leave more concave scars, producing very low edge angles. Similarly, handaxe refinement (width/thickness) correlates with convexity, likely explained by thinner handaxes requiring a greater investment in flaking, and this increased flaking resulting in many small flake scars that contribute to a slightly convex surface. Next, the scatter plot of transverse asymmetry versus edge angle shows that sharper edges tend to be slightly more asymmetrical in transverse view. This likely reflects the greater difficulty in making the minor adjustments needed to shift the plane of intersection between the two handaxe faces when the handaxe is thinner and lower angled. Lastly, transverse asymmetry and convexity/concavity are related also, with more convexity being weakly correlated with less asymmetry. The large flake removals that generate large areas of concavity often also skew the edge towards one face of the biface away from the other, thus raising the amount of transverse asymmetry. Meanwhile, slightly convex edge surfaces typically involve a great investment in attention, being produced via many small removals (e.g. edge trimming). This investment in preparing handaxe edges appears to be also geared towards achieving more symmetrical edges in transverse view.

## Discussion

For the first time, the CEAM method allows for the detection, segmentation, and measurement of the continuous outlines of tool edges. This method is not limited to handaxes alone, but could be applied to any lithic or non-lithic tool. Importantly, every measurement stage of this 3D analysis is entirely automatic, with no user-input required, as well as customizable and can be tailored to any artifact type. Entire assemblages can be analyzed at once, with data being automatically generated. Artifact edges can be automatically identified, measured, and classified without the biases associated with user-input.

We tested the utility of this computational 3D toolkit on a sample of 3D models of handaxes from the later Acheulean southern Levant, finding many handaxes with blunt portions of perimeter, and even many handaxes with multiple sharp edges. The prevalence of handaxes with blunt portions of edge has been increasingly acknowledged over the last few decades, especially in the Levant. At Kissufim for instance, Ronen et al.^23^ needed to add three categories to Bordes’^83^ handaxe type list to accommodate the presence of handaxes with blunt bases, 6% with entirely cortical bases and 10% that were worked but not sharp. The relatively high prevalence of such handaxes has also been noted at Ma’ayan Barukh^24,25^, Tabun^22^, Revadim^21^, and Holon^20^. We confirm here the pervasiveness of handaxes with areas well-suited to grasping, with 63.7% of handaxes in our sample possessing a blunt portion of perimeter, at a rate even higher than previously estimated. Moreover, we offer a repeatable way of more objectively delineating sharp from non-sharp portions of edge so that this variable can be similarly computed at other sites.

Beyond the prevalence of blunt edges in our sample, we also found many handaxes possessed multiple sharp edges that could be statistically distinguished. Our finding that 34.7% of handaxes possessed more than one possible use-edge corresponds with traditional techno-functional analyzes, where differences in edge geometry can lead to the identification of bifaces with more than one edge^15,43,50–53^. Such pieces conform to Boëda’s^84^ notion of a biface as a blank for tools—“*la pièce bifaciale support d’outil*”^84^ p.63, as opposed to a biface as a tool—“*la pièce bifaciale/outil*”^84^ p.64. Handaxes in the biface-as-a-blank-for-tools category potentially possess multiple tools applied via retouch to different portions of the same item. As these different edges may potentially be better suited to fulfill different functions, these handaxes may also confer a greater level of potential for multi-functionality. Here, we provided an automatic and objective means of helping to differentiate bifaces that represent a single tool versus those that potentially represent multiple tools. We hope the methods presented here could be used to help in the performance of these techno-functional analyzes.

Above all, these results reveal a remarkable level of variability in the nature of handaxe edges. The handaxes from our case study are of similar sizes and overall shape^19^, belonging to the same region and sub-period of the Acheulean. Despite these similarities, our results show that handaxes in this period and region are diverse in the number of discrete edges they possess, as well as in their relative length, sharpness, and edge surface concavity.

Among our sample, the edges of the handaxes from Ma’ayan Barukh and Nahal Zihor stand apart from Holon and Jaljulia especially. Those at Holon and Jaljulia possess more discrete edges, a greater proportion of blunt perimeter, and edges with more concavity. Interestingly, Ma’ayan Barukh and Nahal Zihor stand out from the other sites in terms of their location, at the northern and southern extremes of this study area, as well as in the Rift Valley instead of the coastal plain. Raw material quality may partly explain this inter-site variability, as the raw material used to make handaxes at these two sites is almost exclusively cryptocrystalline flint^18,85,86^, as opposed to smaller cobbles of brecciated flint as is commonly used at sites on the coastal plain^21,87–89^. However, subtle differences in flint quality are unlikely to constrain the amount of knapped perimeter or number of discrete edges, features that are typically more functionally determined.

Site function is another possible explanation, as Ma’ayan Barukh and Nahal Zihor are both surface scatters, as opposed to in situ excavations and locales of recurrent occupation, like at Holon, Jaljulia, and Revadim^20,90,91^. Most lithic artifacts recovered from Ma’ayan Barukh and Nahal Zihor were handaxes^85,86^, in stark contrast with Holon, Jaljulia, and Revadim where handaxes are merely a component of a more diverse toolkit. At each of these sites, handaxes were less than 5% of the lithic artifacts larger than 2 cm^21,90–92^. It is possible that the greater diversity of tasks undertaken at these more routinely occupied sites may have necessitated the greater diversity of handaxe edges. A variety of combinations of sharp and blunt handaxe segments increases the potential for multifunctional use of these artifacts. The methods presented here open new ways of exploring handaxe multifunctionality in the Acheulean and technological variability more broadly.

## Conclusion

This new method for detecting, segmenting, and measuring handaxe outlines moves us towards more accurate and repeatable measures of handaxe techno-morphological variability. The question of what constitutes the ‘edge’ of a handaxe is a complex and ambiguous one. We put forth an automatic and objective method of making this differentiation, subsequently allowing for the precise measurements of several previously difficult to measure variables, like length, angle, transverse asymmetry, and surface concavity. The issue of lithic edge angles is a similarly fraught question. The edge angle of any object with complex geometry is a question less about how the angle is measured, and more about what is considered an ‘edge’. For this reason, there is no correct edge angle value for a single handaxe. How we measure an edge angle relates to what we define as an edge. Continuous methods allow us to incorporate as much of the edge into this definition as we wish. We hope to have offered a step forward in the continuous measurement of lithic artifacts. While many traditional analysis methods render single values or categories from the complex geometry of lithic artifacts, this complex geometry makes many of their techno-morphological variables continuous, rather than discrete. Novel ways of computing this continuous variability has much potential to reveal subtle and previously overlooked lithic variability.

This techno-morphological variability is fundamental to interpretations about past hominin behavior, decision making, skills, and cognition. Making these interpretations necessitates a firm understanding of tool geometry. Thus far however, the variability of handaxe edges has been sorely understudied. The majority of studies on handaxes explore their 2D plan asymmetry, their refinement (width/thickness), and their overall morphology, either via shape indices or via 3DGM methods. It is clear from our analysis however, that these approaches overlook significant portions of handaxe variability. Even among handaxes of similar morphology, their edges can be markedly variable. This variability has likely been largely overlooked due to the difficulty in measuring edges reliably. As such, when handaxe edges have been addressed, this has largely been done qualitatively or with unreliable methods of measurement. Here, we have taken a step towards making these features more readily quantifiable. We hope this toolkit will assist in the more objective, repeatable, and automatic measurement of key variables of tool-edge geometry.

## Materials and methods

### Sample

**Figure 7:**
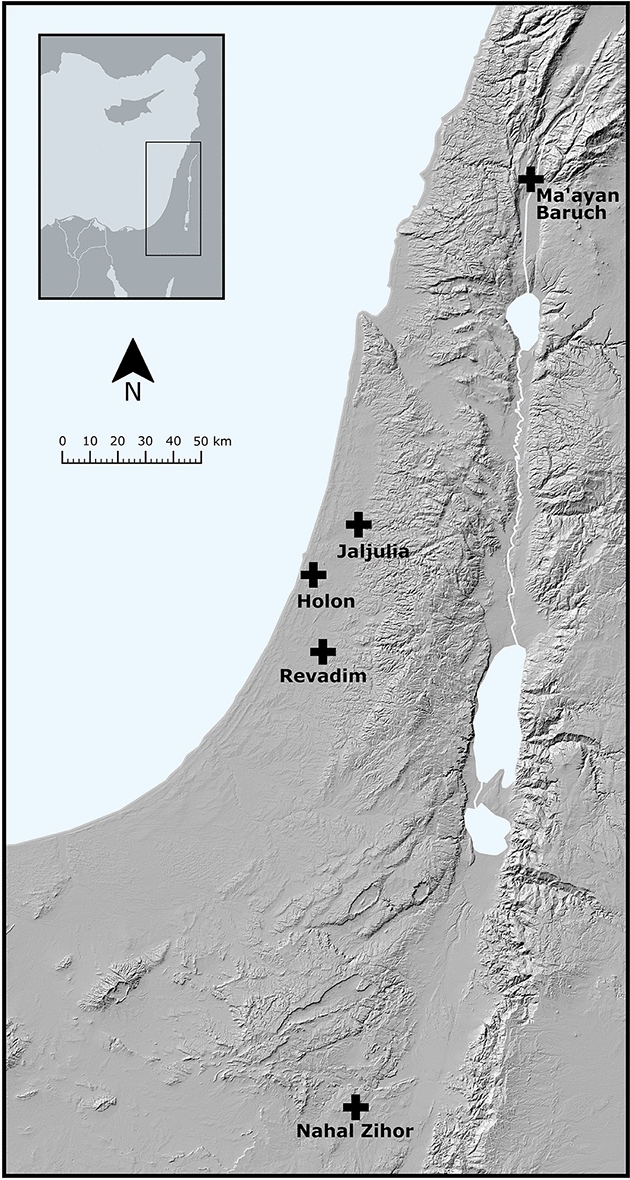
Map of the five later Acheulean sites analyzed here.

For this analysis, 3D scans were acquired for 686 handaxes from five sites, either from an open-access repository^93^ or via scanning with a structured light 3D scanner (following methods outlined in^7,94–97^). The five sites analyzed here (from North to South) are Ma’ayan Barukh (N = 160), Jaljulia (N = 260), Holon (N = 75), Revadim (N = 60), and Nahal Zihor (N = 131) (Fig. 7). All sites are open-air sites, chosen due to the large number of handaxes available, and are ascribed to the later Acheulean based on a mixture of absolute and relative dating methods^64,85,86,90,91,98–102^. Ma’ayan Barukh and Nahal Zihor are surface scatters, the remainder are excavated sites on the coastal plain of the southern Levant. Broken and damaged handaxes were excluded from this sample, except in cases where any post-depositional damage was very minor, barely affecting the outline or shape of the handaxe. All 3D models of handaxes analyzed here are available on an open access online repository uploaded for this present study (N = 533)^81^ and on a repository (N = 153)^93^ from a previous analysis by Herzlinger et al^19^.

### Pre-processing in Artifact3-D

Prior to conducting the CEAM analysis, a couple of pre-processing steps were first undertaken on the 3D models in *Artifact3-D*^38,103^, software designed for user-friendly manual analysis of 3D models. The wrl/vrml 3D models were imported into the program and consistently oriented using the *Create Qins File* and *Normal Positioning* functions respectively. These steps generate a .mat file that stores the vertices and faces of the 3D model along with the rotation matrix corresponding to the automatic or manual orientation chosen by the user. The following steps can then be undertaken automatically, entirely without user-input, by loading this .mat file and running the MATLAB script available in the online repository^81^.

### Outline detection

The first step of this automatic analysis involves identifying the 3D perimeter of each handaxe, dividing them into their two hemispheres or faces. First, the 2D outline of each handaxe was found by identifying the edges of its alpha shape (the polygon that bounds a set of coordinates), essentially finding the most extreme coordinates of the handaxe in x–y space^104,105^. Combining the z coordinates of the handaxe’s 3D mesh corresponding to these extreme x–y coordinates gave a set of 3D outline coordinates.

The coordinates comprising the outline were normalized to the same number of coordinates (1000) for each handaxe. This value was chosen as it is just below the handaxe in our sample with the least number of edge coordinates. The edge coordinates were reduced to this equal number of coordinates for each handaxe using linear interpolation, maintaining the relative spacing between coordinates so as to not distort the outline. Similarly, the entire 3D mesh was also normalized to the same number of coordinates (50,000) to avoid the confounding influence of scan resolution. This step was necessary as variable levels of mesh density could slightly influence the edge angle measurements, with lower resolutions resulting in slightly higher angle values. A relatively low number of coordinates was chosen so that 3D models made with lower resolution scanners would produce comparable results.

The variable of size was also controlled by scaling each handaxe to the same length, with each other dimension scaled proportionally to maintain their original 3D shape. This results in all handaxes having a length of 1, while their 3D shape remains unchanged. By both standardizing resolution and resizing the handaxes before analysis, each handaxe is normalized, making them comparable without the influence of unwanted confounding variables.

For handaxes that are sharp around their entire perimeter, this method was sufficient to find the handaxe outline. But, for handaxes with un-knapped and/or blunt portions of outline, the z coordinate of this outline tended to deviate wildly over these areas as they are often perpendicular to the x–y plane (Fig. 1a). Thus, we needed to find these peripheral perpendicular surfaces and find a fair way to redefine the outline in these areas. To do so, we computed how parallel the normal vector of each triangle of the 3D mesh was to the x–y plane (Fig. 1b). Then, we took these values closest to the outline, resulting in a continuous measure of the normal vectors of the tool’s outline (Fig. 1c). Where the outline normals were near-parallel to the x–y plane (orange or red in Fig. 1b, c), the z value of the outline was changed to the average of the z values of coordinates within a k-nearest neighborhood (Fig. 1d). Meanwhile, the x and y coordinates remained unchanged. This process resulted in a reliably detected handaxe outline, without major fluctuations in outline position due only to blunt portions of the outline interfering with the projection.

### Continuous Edge Angle Measurement (CEAM)

The continuous nature of handaxe edges necessitates a continuous measurement. Thus, we introduce CEAM. The first step was to compute the edge angle of every coordinate of the handaxe’s 3D model. Each 3D model of a handaxe is comprised of thousands of individual xyz coordinates, in this case normalized to 50,000 coordinates. Any angle measurement requires 3 points. Thus, for each individual coordinate of the 3D mesh, we found two other coordinates. The first was the coordinate on the opposing face of the handaxe that was closest in x–y space (i.e. possessing the closest x and y values). The second was the closest coordinate on the outline (Fig. 2a). We then considered the two vectors between the outline coordinate and the two face coordinates and computed the vector magnitude of the dot and cross products of these two vectors, giving an edge angle value. This process is repeated iteratively for all coordinates of the 3D mesh (Fig. 2b).

This process gave an angle value to every coordinate of the handaxe. As we were only interested in the angle of the edge itself, we limited our calculation to the outer 20% of the handaxe’s surface area (the inner black line of Fig. 2c). If desired, this arbitrary cut-off can be altered to match the tool-type being analyzed. To ensure that this 20% value was not unduly influencing the edge angle results, we re-ran the CEAM analysis at different proportions of surface area. The plot on the right of Fig. 2c shows the mean edge angle for this example handaxe plotted with different surface area percentages (1–25%). Once the percent of surface area was larger than 10%, the mean edge angle value converged, with our chosen 20% value sitting on this plateau (marked with an x). Thus, the influence of changing this surface area percent is negligible.

Next, for each coordinate of the outline, we found its k-nearest neighbor coordinates and took the maximum kernel density of these values, analogous to the most frequently occurring edge angle (Fig. 2c). This method excludes outliers from the angle calculation. These outliers commonly occur on the most peripheral coordinates of the scan where the angle can approach 180° due to post-depositional rounding or beveling and the resolution limits of 3D scanners. Although k-nearest neighbors introduces a small amount of smoothing, excluding these outliers is necessary, as taphonomic rolling has been shown to influence handaxe outlines^106^, and including these artificially higher edge angle values would skew the average angle values. This process was repeated for every outline coordinate, giving a CEAM for the entire handaxe perimeter (Fig. 2d).

To ground truth this method and ensure its accuracy and precision, we ran the CEAM analysis on a regular octahedron (Fig. 2d). Its mean edge angle value of 109.47° (SD = 0.005) matches its known dihedral angle (arccosine of − 1/3, or 109.47°). Any further comparisons with previous angle measurement methods, manual or digital, are difficult due to the difference between discrete and continuous metrics. Each edge angle measurement is comprised of a simple calculation involving three coordinates in Euclidean space. The difficulty lies in choosing how these three coordinates are selected and how many sets of three coordinates are retained. Thus, we measured the edge angle for every coordinate of each handaxe’s 3D scan, involving thousands of angle calculations. This makes comparisons with previous methods of angle measurement impossible, as they mostly involve single, or a very small number of, angle calculations, with user-defined and manually selected coordinates a certain distance from the edge. Any future comparisons with new digital and continuous methods will center on the question of how the edge is defined, governing which calculations are retained.

### Edge segmentation

With the edge angle of every handaxe outline coordinate calculated, we then segmented the outline, where possible, based on discontinuities in these angle values. This is aimed at revealing portions of handaxe perimeter that are sufficiently sharp to be considered an edge, as well as any edges that possess sufficiently distinct edge angle values to be considered as more than one edge. To do so, we used change point detection, which identifies significant changes in sequential data. The arc length, starting at the tip (maximum y value), was plotted against the edge angle values of the handaxe perimeter, as seen in the plots of Fig. 3. Then, the edge angle values were segmented according to the location/s along the arc length that minimize the sum of the residual error around each segment’s mean value. These most significant changes are called change points and visualized with red vertical lines in Fig. 3, while the local mean of each segment is shown with blue horizontal lines. For the example handaxe in Fig. 3, we show the result of different numbers of change points (0–8) as examples of the possible ways of segmenting the perimeter of an individual handaxe.

Even visually, it is clear that 0 change points is insufficient to summarize the variability of edge angle values around the perimeter, while 6 or more change points are ‘over-fitting’ the data. To make this selection objectively and automatically, consider the amount of residual error, or the distance between the edge angle values (black lines) and the mean value (blue horizontal lines). With each additional change point added, the residual error decreases. But the decrease in residual error is more dramatic when adding the first few change points, and this decrease decays roughly exponentially. Thus, when we plot the gradient of residual error against the first 10 change points (Fig. 3), the ‘elbow’ of this plot (shown by a green x), represents the point at which any additional change points will reduce the residual error only marginally. The location of this ‘elbow’, in this case at 3 change points, provides an automatic way of choosing how many segments with which to divide each handaxe’s perimeter. For this example handaxe then, only three change points were needed, matching a visual examination of the outline which shows a yellow segment (approximately 160°), a light blue segment (approximately 90°), and a dark blue segment (approximately 60°). This is further confirmed by the histogram (Fig. 3), showing the frequency of edge angle values, with three peaks in the distribution for this example handaxe. Those with only one segment should have only one peak, those with two segments should have two peaks, and so on. Importantly, this segmentation, including the selection of number of segments, was done entirely automatically, free from user-input. More examples are shown in Fig. 3b, where this method of segmenting handaxe outlines according to edge angle values reliably and automatically found the discrete outline segments of these handaxes.

### Edge segment measurements

Having automatically segmented each handaxe into discrete outline portions, we now seek to measure these portions individually. Edge length was precisely measured by summing the 3D distances between each consecutive outline coordinate. Using the automatic edge segmentation procedure described above, we compared the edge length of the sharp portion of the outline versus the blunt portion. Importantly, each handaxe’s scale and resolution was normalized, as the scale at which length measurements are taken influences the length itself, whether it is the scale at which coastlines are measured^107^, or lithic edges are measured^29^.

**Figure 8:**
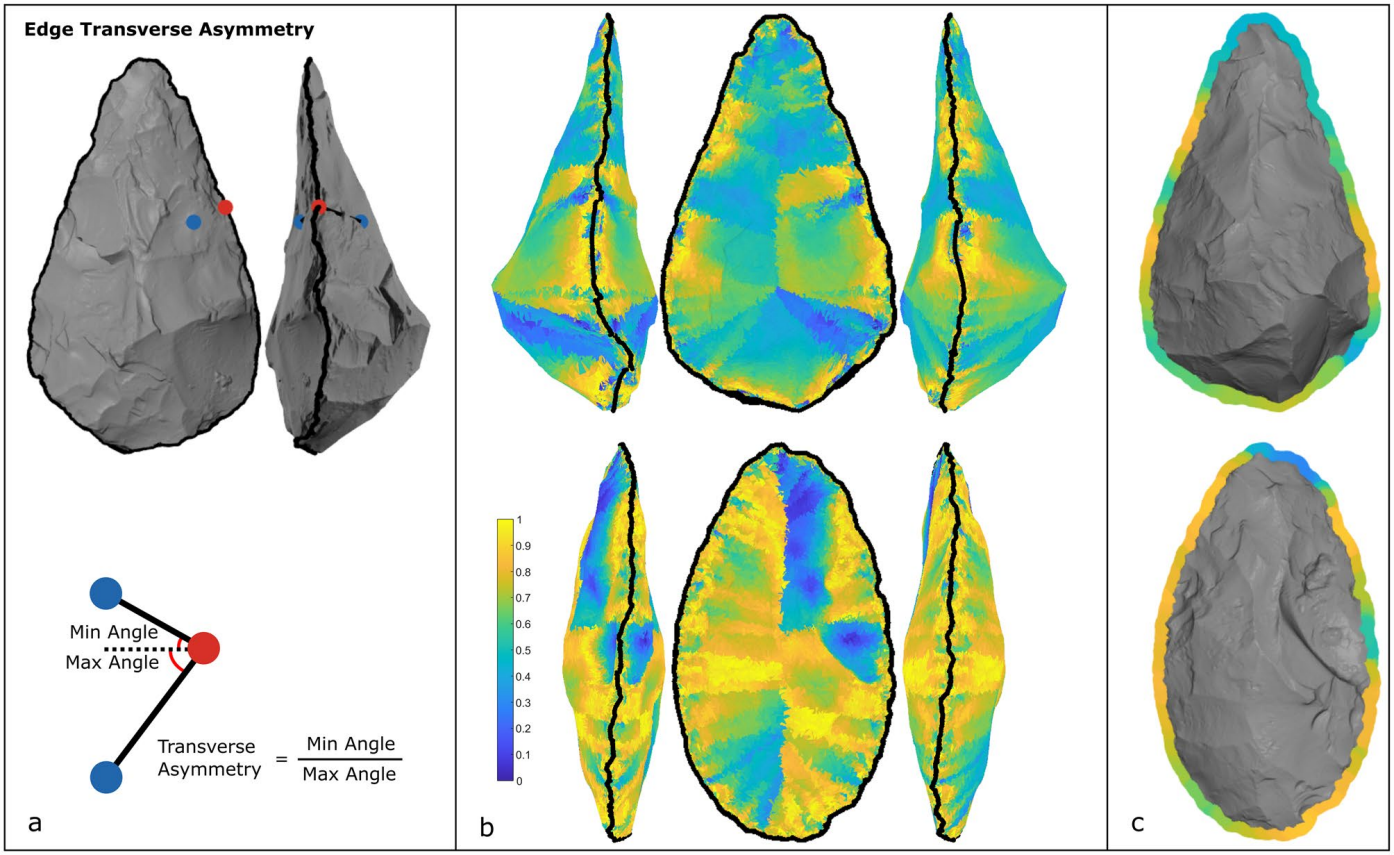
Edge transverse asymmetry method. (**a**) Each coordinate of the 3D mesh is compared with its nearest coordinate on the opposing face of the biface (in x–y space) and the closest coordinate on the outline, blue and red dots respectively. The ratio between these angles serves as a measure of transverse edge asymmetry. (**b**) This process is repeated for every coordinate of the 3D mesh. (**c**) As in the CEAM method detailed above, only the values for the coordinates in a k-nearest neighborhood are used to provide continuous transverse asymmetry values around the entire perimeter of each handaxe. Higher values (yellow) represent less asymmetry, while lower values (blue) denote more asymmetry. Two example handaxes are shown, one that is plano-convex (top) and one whose edges are much less asymmetric (bottom). Note the slight twist in the outline of the lower handaxe, where even though its edges mostly possess low asymmetry, areas near its tip and base are markedly asymmetric.

We also measured edge transverse asymmetry, or the amount of asymmetry along the edge in section view. Plano-convex handaxes, for instance, possess very asymmetrical edges in transverse view^108–111^. We computed the amount of edge transverse asymmetry for each 3D coordinate of the outline by separating the edge angle by the vector that extends from the edge coordinate towards to the plan-view center of the artifact, parallel to this plan view (i.e. the x–y plane). Handaxe edges with low transverse asymmetry possessed relatively equivalent angle values either side of the vector parallel to the x–y plane, while plano-convex handaxes possessed high transverse asymmetry (Fig. 8). We computed transverse asymmetry as the ratio between the minimum and maximum angle either side of this vector. Thus, transverse asymmetry values approached 1 where both sides of the edge were very similar and approached 0 when they are very asymmetrical.

**Figure 9:**
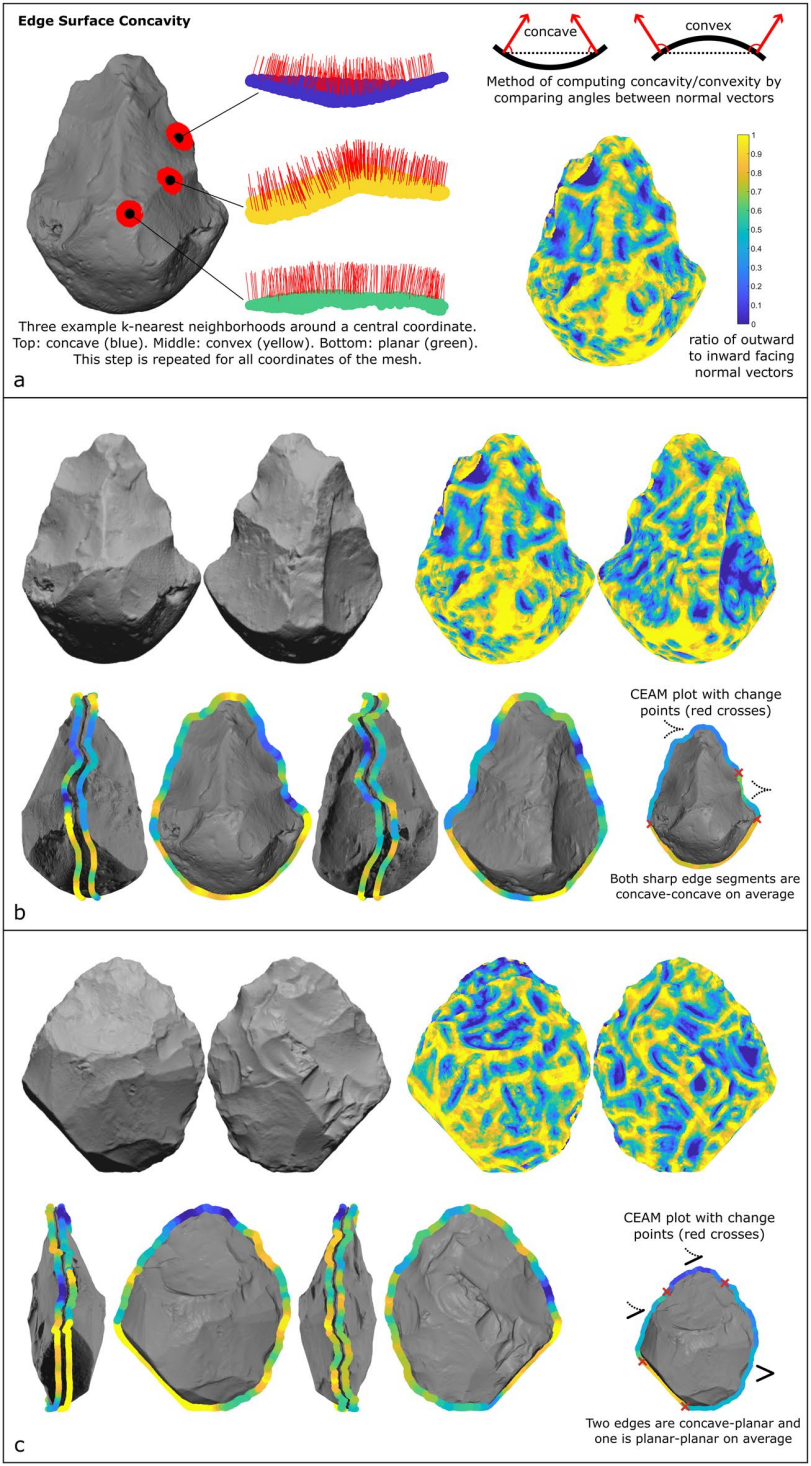
Edge surface concavity method. (**a**) The method for measuring concavity for each individual coordinate of the 3D mesh, using the direction of the normal vectors in a k-nearest neighborhood around each coordinate. The ratio of outward to inward facing normal vectors serves as a quantification of concavity versus convexity. (**b**) An example handaxe, with concavity values for the entire mesh surface (top) and for each coordinate of the handaxe outline (bottom) identified in the same fashion as the CEAM method above. The surface concavity of the outline is calculated for both faces of the handaxe, resulting in the parallel outlines shown in the side views of the handaxe. Combining the CEAM analysis with the concavity analysis shows this example handaxe has two sharp edges that are both concave-concave. (**c**) Another example handaxe. The combined CEAM and concavity analysis reveals that this example handaxe has two concave-planar edges, one moderately sharp and one sharper edge segment, as well as one planar-planar moderately sharp edge.

Lastly, for each discrete edge segment, we computed the concavity of the surface near the edge. Surface concavity/planarity/convexity was calculated by comparing the normal vectors in a k-nearest neighborhood around each individual coordinate. Normal vectors that point towards each other indicate concavity, vectors that point away from each other indicate convexity, and parallel vectors indicate planarity (Fig. 9a). For speed, only the outermost normal vectors within this neighborhood were compared to the central coordinate. Values for concavity were given as the ratio of normal vectors that point away from the center normal versus those that point towards it, with 0 representing entirely concave surfaces and 1 representing entirely convex surfaces. As with the CEAM method, the most frequently occurring concavity values nearest to each outline coordinate (outermost 20% of the handaxe’s surface area), gave a continuous measure of concavity to the handaxe perimeter (Fig. 9b, c).

## Data Availability

All 3D models used in this study are available on the following open-access repositories: Herzlinger, G. et al. Reevaluation of the classification scheme of the Acheulian in the Levant–50 years later: A morpho-technological analysis of handaxe variability. Open Sci. Framew. https//osf.io/yz7k3/ (2020) 10.17605/OSF.IO/YZ7K3. Muller, A. & Grosman, L. 3D models and code for the analysis of later Acheulean southern Levantine handaxes. https://zenodo.org/records/10420897 (2023) 10.5281/zenodo.10420897. The code used in this analysis is available at the latter repository. The results generated by this code are also available from the corresponding author (AM) upon request.
